# Alternating patterns of seasonal influenza activity in the WHO European Region following the 2009 pandemic, 2010‐2018

**DOI:** 10.1111/irv.12703

**Published:** 2020-01-16

**Authors:** Piers Mook, Tamara Meerhoff, Sonja J. Olsen, René Snacken, Cornelia Adlhoch, Dmitriy Pereyaslov, Eeva K. Broberg, Angeliki Melidou, Caroline Brown, Pasi Penttinen, Collective of the WHO European Region, European Influenza Surveillance Network, Artan Simaku, Iris Hasibra, Liana Torosyan, Shushan Sargsyan, Monika Redlberger‐Fritz, Judith H Aberle, Oleg Salimov, Nazakat Abdullayeva, Veronika Shimanovich, Natalia Gribkova, Nathalie Bossuyt, Isabelle Thomas, Anna Kurchatova, Neli Korsun, Goranka Petrović, Vladimir Draženović, Maria Koliou, Despo Pieridou, Martina Havlickova, Jan Kyncl, Tyra Grove Krause, Ramona Trebbien, Olga Sadikova, Natalja Kuznetsova, Vincent Enouf, Sibylle Bernard‐Stoecklin, Niina Ikonen, Outi Lyytikäinen, Ann Machablishvili, Khatuna Zakhashvili, Silke Buda, Ralf Dürrwald, Ourania Kalkouni, Georgia Gioula, Zsuzsanna Molnár, Mónika Rózsa, Linda Dunford, Joan O Donnell, Gudrun Sigmundsdottir, Gudrun Erna Baldvinsdottir, Zalman Kaufman, Michal Mandelboim, Caterina Rizzo, Maria Rita Castrucci, Sultanova Meirim, Altynay Sagimbay, Otorbaeva Dinagul, Bodoshev Azat, Raina Nikiforova, Natalija Zamjatina, Asta Skrickienė, Algirdas Griškevičius, Guillaume Fournier, Joel Mossong, Jackie M Melillo, Graziella Zahra, Božidarka Rakočević, Zoran Vratnica, Gé Donker, Adam Meijer, Golubinka Bosevska, Vladimir Mikikj, Trine Hessevik Paulsen, Olav Hunges, Lidia B Brydak, Katarzyna Luniewska, Raquel Guiomar, Ana Paula Rodrigues, Stefan Gheorghita, Constantin Spinu, Rodica Popescu, Alina Ivanciuc, Anna A Sominina, Elena Burtseva, Dragana Dimitrijevic, Svetlana Filipovic‐Vignjevic, Arijana Kalaveshi, Maja Sočan, Katarina Prosenc, Ivan Bakoss, Edita Staroňová, Amparo Larrauri, Francisco Pozo, AnnaSara Carnahan, Mia Brytting, Damir Perisa, Ana Rita Gonçalves, Tamanno Safarova, Niginamo Zakirova, Emine Avci, Ayse Basak Altas, Oksana Artemchuk, Iryna Demchishyna, Richard Pebody, Joanna Ellis, Conall McCaughey, Mark O'Doherty, Jim Mcmenamin, Arlene Reynolds, Simon Cottrell, Catherine Moore, Sultana Djemileva, Ravshan Rakhimov, John McCauley, Rod Daniels

**Affiliations:** ^1^ Division of Health Emergencies and Communicable Diseases High Threat Pathogens WHO Regional Office for Europe Copenhagen Denmark; ^2^ Radboud University Medical Center Radboud Institute for Health Sciences Department of Primary and Community Care Nijmegen The Netherlands; ^3^ European Centre for Disease Prevention and Control (ECDC) Stockholm Sweden

**Keywords:** Central Asia, Europe, influenza, surveillance

## Abstract

**Background:**

Influenza virus infections are common and lead to substantial morbidity and mortality worldwide. We characterized the first eight influenza epidemics since the 2009 influenza pandemic by describing the distribution of viruses and epidemics temporally and geographically across the WHO European Region.

**Methods:**

We retrospectively analyzed laboratory‐confirmed influenza detections in ambulatory patients from sentinel sites. Data were aggregated by reporting entity and season (weeks 40‐20) for 2010‐2011 to 2017‐2018. We explored geographical spread using correlation coefficients.

**Results:**

There was variation in the regional influenza epidemics during the study period. Influenza A virus subtypes alternated in dominance, except for 2013‐2014 during which both cocirculated, and only one season (2017‐2018) was B virus dominant. The median start week for epidemics in the Region was week 50, the time to the peak ranged between four and 13 weeks, and the duration of the epidemic ranged between 19 and 25 weeks. There was evidence of a west‐to‐east spread across the Region during epidemics in 2010‐2011 (*r* = .365; *P* = .019), 2012‐2013 (*r* = .484; *P* = .001), 2014‐2015 (*r* = .423; *P* = .006), and 2017‐2018 (*r* = .566; *P* < .001) seasons. Variation in virus distribution and timing existed within reporting entities across seasons and across reporting entities for a given season.

**Conclusions:**

Aggregated influenza detection data from sentinel surveillance sites by season between 2010 and 2018 have been presented for the European Region for the first time. Substantial diversity exists between influenza epidemics. These data can inform prevention and control efforts at national, sub‐national, and international levels. Aggregated, regional surveillance data from early affected reporting entities may provide an early warning function and be helpful for early season forecasting efforts.

## 1 INTRODUCTION

Influenza virus infections are common and lead to substantial morbidity and mortality^1^ worldwide. Influenza surveillance remains critically important to public health as influenza viruses constantly change and pose a continued risk of a novel virus emerging and causing a pandemic. Influenza surveillance is conducted nationally (sometimes sub‐nationally), supra‐nationally, and globally with a range of objectives which include determining when and where influenza activity is occurring, identifying the circulating influenza type, A subtype and B lineage, detecting changes in the antigenic and genetic characteristics of seasonal influenza viruses to inform the composition of influenza vaccines biannually,^2^ and describing the clinical patterns of influenza.

The WHO European Region comprises 53 Member States (formally recognized countries by WHO, including 28 in the European Union, 24 elsewhere in Europe and Central Asia, and Israel) and 900 million inhabitants.^3^ Influenza surveillance in Europe was first established under the Eurosentinel project and European Influenza Surveillance Scheme and has been coordinated by the WHO Regional Office for Europe and the European Centre for Disease Prevention and Control (ECDC) since 2008. Fifty Member States and Kosovo1In accordance with Security Council resolution 1244 (1999). routinely collect influenza surveillance data and report it to The European Surveillance System (TESSy) hosted at ECDC. Collated data on influenza activity for the Region are analyzed and, since 2014, reported weekly in the joint Flu News Europe bulletin during the influenza season.^4^

To further inform evidence‐based decision making for influenza preparedness and control, we characterized the first eight influenza seasons following the 2009 influenza pandemic in terms of virus distribution by type, subtype and lineage, age, timing, and geographical spread.

## 2 METHODS

### 2.1 Study design

We conducted a retrospective analysis of laboratory‐confirmed influenza detections from sentinel surveillance of persons seeking care for a respiratory illness in an ambulatory setting (ie, seeking care at a general practitioner or primary healthcare facility). We included data from 50 Member States in the WHO European Region which corresponded to 54 reporting entities with data from England, Northern Ireland, Scotland, and Wales (all in the United Kingdom) and Kosovo^1^ reported separately. We present data by reporting entity.

### 2.2 Study population and data

Sentinel influenza surveillance is conducted in a representative subset of outpatient sites and coordinated by national (and occasionally sub‐national) networks; samples should be collected from patients using a systematic sampling scheme with pre‐defined influenza‐like illness (ILI) and/or acute respiratory infection (ARI) case definitions. Reporting entities collected and reported sentinel influenza surveillance data to TESSy each week: the number of patients seen or the population in the catchment area of sites; the number of patients presenting with ILI and/or ARI; and, of these, the number of samples collected and tested for an influenza virus, and the test results. National influenza surveillance systems and case definitions varied by reporting entity.^5^ Influenza virus detection was typically by reverse transcription polymerase chain reaction.^6^

Reporting entity‐week data for the period 2010 to 2018 were extracted from TESSy in August 2018. Influenza season was defined for the northern hemisphere as ISO week^7^ 40 in a given year to ISO week 20 in the following year. Reporting entity‐week data from earlier than week 40/2010, later than week 20/2018 or not attributed to season weeks were excluded.

### 2.3 Analyses

For regional aggregated analyses, the distribution of all available virological data derived from specimens taken from ILI or ARI cases in sentinel outpatient sites was summarized by influenza type, influenza A subtype, influenza B lineage, and season. The dominant circulating virus was defined for each season and system as ≥60% of influenza viruses, subtyped type A viruses and type B viruses with lineage, and codominance as the proportion of viruses circulating between 41% and 59%. For reporting entity‐level analyses, data for a given reporting entity were excluded if, for a particular season, there were fewer than 50 specimens or <20 weeks of data submitted to TESSy. Ranges of seasonal, reporting entity level data were only described where there were at least five seasons of valid data, as per these criteria. Untyped influenza viruses, influenza A viruses not subtyped, and influenza B viruses not ascribed to a lineage were not included in the denominator for type, subtype, and lineage percentage calculations, respectively, for aggregated regional and reporting entity‐level analyses.

Temporal analyses were conducted using the percentage of specimens taken in sentinel outpatient sites testing positive for an influenza virus (ie, percent positive) with aggregate data for all reporting entities in the region and by reporting entity. Timing of the start of the epidemic at both regional and reporting entity levels for a season was defined as the first of two consecutive weeks with at least 10% positive specimens and the end of the epidemic as the last week with a percent positive of at least 10% or week 20. The duration of the epidemic was defined as the number of weeks between the start and end weeks, inclusive. The peak of the epidemic was defined as the week with the highest percent positive. The time from the start to the peak of the epidemic was defined as the number of weeks between the start and peak weeks, exclusive. The number of weeks with high influenza circulation during an epidemic was calculated as those with at least 40% positive (a threshold that is used operationally in routine regional surveillance practices and based on expert opinion). For percent positive to be valid, at least 10 specimens had to have been tested in a given week per reporting entity. Peak and end weeks and number of weeks above 40% positive were only valid if there was a prior epidemic start week defined.

To explore spread of influenza infections across the Region within a season, we calculated the correlation coefficient between the timing of the start week of the epidemic in a reporting entity and the reporting entity's geographical center defined as the rounded latitude and longitude of the geographical center of each reporting entity in decimal degrees.^8^ A correlation (*r*) of 0‐.19 was defined as very weak, .20‐.39 as weak, .40‐.59 as moderate, .60‐.79 as strong, and .80‐1 as very strong.^9^ The threshold of significance was set at the 5% level and derived by applying linear regression to these data. Reporting entities with no defined epidemic start date were excluded from these analyses.

**Figure 1:**
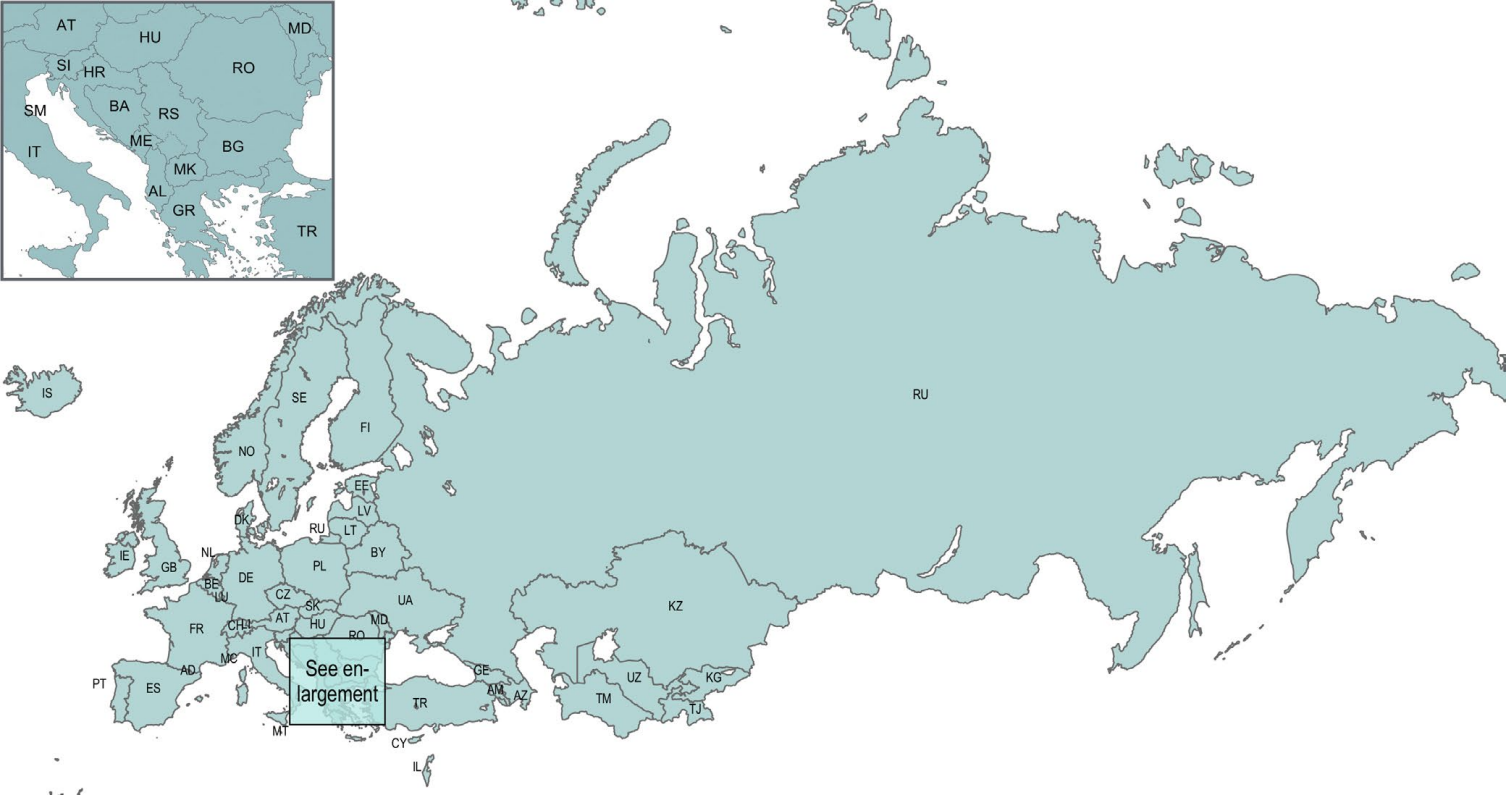
Map of member states of the WHO European Region. AL: Albania, AD: Andorra, AM: Armenia, AT: Austria, AZ: Azerbaijan, BY: Belarus, BE: Belgium, BA: Bosnia and Herzegovina, BG: Bulgaria, HR: Croatia, CY: Cyprus, CZ: Czech Republic, DK: Denmark, EE: Estonia, FI: Finland, FR: France, GE: Georgia, DE: Germany, GR: Greece, HU: Hungary, IS: Iceland, IE: Ireland, IL: Israel, IT: Italy, KZ: Kazakhstan, KG: Kyrgyzstan, LV: Latvia, LT: Lithuania, LU: Luxembourg, MT: Malta, MC: Monaco, ME: Montenegro, NL: Netherlands, NO: Norway, PL: Poland, PT: Portugal, MD: Republic of Moldova, RO: Romania, RU: Russian Federation, SM: San Marino, RS: Serbia, SK: Slovakia, SI: Slovenia, ES: Spain, SE: Sweden, CH: Switzerland, TJ: Tajikistan, MK: North Macedonia, TR: Turkey, TM: Turkmenistan, UA: Ukraine, GB: United Kingdom of Great Britain and Northern Ireland, and UZ: Uzbekistan. Note: The boundaries and names shown and the designations used on this map do not imply the expression of any opinion whatsoever on the part of the World Health Organization concerning the legal status of any country, territory or area or of its authorities, or concerning the delimitation of its frontiers or boundaries. Dotted lines on maps represent approximate borderlines for which there may not yet be full agreement

We conducted two sensitivity analyses: (a) given substantial geographical span across the Region, we removed the Russian Federation and re‐ran the geographical correlation analysis to identify any changes (Figure 1); and (b) we restricted data up to and including the regional epidemic start week each season to see whether the regional virus distribution at that time point was the same or different to that seen when looking retrospectively back on a full season. Reporting entity‐specific summary data are available in Table S1.

Analyses were conducted in Microsoft Excel 2013, OpenEpi,^10^ R (R Core Team [2018]; R Foundation for Statistical Computing, Vienna, Austria) and Stata 14 (StataCorp). Percentages were calculated and the median and range used to summarize data. The chi‐squared or Fisher's exact tests were applied to examine equality of proportions and linear regression models used to investigate the relationship between variables and time at regional and national levels.

## 3 RESULTS

**Figure 2:**
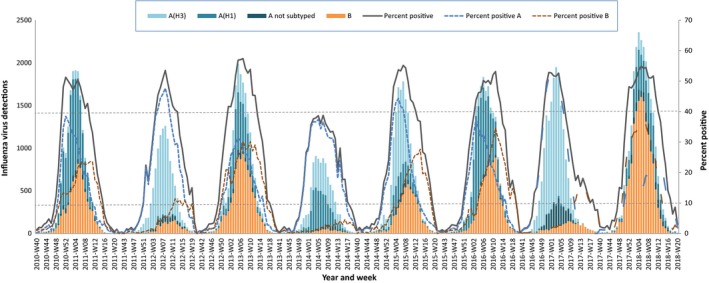
Number of influenza viruses by type and subtype and percentage of specimens positive for influenza virus detected from sentinel outpatient surveillance in the WHO European Region by season, 2010‐2018. 10% and 40% positivity thresholds displayed

**Figure 3:**
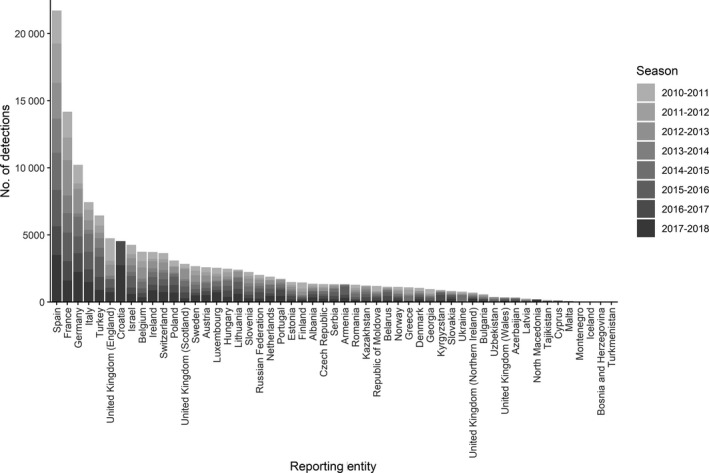
Total number of detections reported from sentinel outpatient surveillance in the WHO European Region by reporting entity and season, 2010‐2018. Data for Kosovo (In accordance with Security Council resolution 1244 (1999)). have not been presented

Between the 2010‐11 and 2017‐18 influenza seasons, 50 reporting entities reported 134 889 influenza detections from cases identified at sentinel outpatient facilities. The number of detections and percent positive by season aggregated across all reporting entities ranged from 9434 (2013‐2014) to 24 875 (2017‐2018) and 25% (2013‐2014; the only instance below 30%) to 41% (2017‐2018), respectively (Table 1). There was no increase in the number of specimens (*P* = .054; linear regression) or detections (*P* = .131; linear regression) over the study period. The maximum weekly percent positive for a season ranged from 39% (2013‐2014) to 57% (2012‐2013) (Table 2 and Figure 2). The total number of influenza detections from the sentinel surveillance system by reporting entities ranged between none (Turkmenistan) and 21,717 (Spain) detections (Figure 3). Six reporting entities, representing roughly 44% of the population of the Region, reported 48% of all detections: Spain (16%), France (11%), Germany (8%), Italy (6%), Turkey (5%), and England (4%).

**Table 1:** Distribution of influenza detections from sentinel influenza‐like illness or acute respiratory infections outpatient surveillance sources by type, subtype, and lineage in the WHO European Region by season, 2010‐2018

	2010‐2011	2011‐2012	2012‐2013	2013‐2014	2014‐2015	2015‐2016	2016‐2017	2017‐2018
No. of reporting entities	43	42	45	43	43	42	45	47

	n (%)	n (%)	n (%)	n (%)	n (%)	n (%)	n (%)	n (%)
Specimens tested	47 046 (100)	37 303 (100)	45 812 (100)	37 003 (100)	43 473 (100)	52 586 (100)	50 865 (100)	60 911 (100)
Influenza detections	17 554 (37)	11 286 (30)	18 205 (40)	9434 (25)	16 079 (37)	19 308 (37)	18 148 (36)	24 875 (41)
Virus distribution^a^								
Influenza A total	10 471 (60)	9774 (87)	9022 (50)	8839 (94)	10 713 (67)	11 166 (58)	16 186 (89)	9215 (37)
A(H1N1)pdm09	8960 (92)	281 (3)	5167 (63)	3919 (47)	2361 (24)	9304 (87)	185 (1)	4985 (65)
A(H3N2)	822 (8)	8815 (97)	3090 (37)	4449 (53)	7630 (76)	1402 (13)	13 585 (99)	2707 (35)
A not subtyped	689	678	765	471	722	460	2416	1523
Influenza B total	7083 (40)	1512 (13)	9183 (50)	595 (6)	5366 (33)	8142 (42)	1962 (11)	15 660 (63)
B/Yamagata	141 (10)	99 (36)	3056 (91)	67 (86)	1330 (97)	148 (4)	480 (56)	7319 (97)
B/Victoria	1223 (90)	179 (64)	306 (9)	11 (14)	42 (3)	3977 (96)	384 (44)	209 (3)
B unknown lineage	5719	1234	5821	517	3994	4017	1098	8132
Availability of A subtype and B lineage data								
% A not subtyped	7%	7%	8%	5%	7%	4%	15%	17%
% B no lineage	81%	82%	63%	87%	74%	49%	56%	52%
Dominant virus type, A subtype and B lineage	A	A	A/B	A	A	A/B	A	B
Dominant A virus subtype	A(H1N1)pdm09	A(H3N2)	A(H1N1)pdm09	A(H1N1)pdm09/A(H3N2)	A(H3N2)	A(H1N1)pdm09	A(H3N2)	A(H1N1)pdm09
Dominant B virus lineage	B/Victoria	B/Victoria	B/Yamagata	B/Yamagata	B/Yamagata	B/Victoria	B/Victoria/ B/Yamagata	B/Yamagata

a For influenza virus type percentage calculations, the denominator was total detections; for A virus subtype and B virus lineage, it was total influenza A viruses subtyped and total influenza B viruses with lineage determined, respectively.

**Table 2:** Timing of start, peak, and duration of influenza season based on percentage of ILI or ARI specimens from sentinel outpatient surveillance positive for influenza viruses in the WHO European Region by season, 2010‐2018

Indicator	2010‐2011	2011‐2012	2012‐2013	2013‐2014	2014‐2015	2015‐2016	2016‐2017	2017‐2018
Start of epidemic week no.	48	51	49	51	51	51	46	48
Start week no. based on A detections only	49	52	51	51	51	51	46	50
Start week no. based on B detections only	50	12	50	a	6	4	11	50
Peak of epidemic week no.	52	8	7	7	7	11	52	5
Time in weeks from start to peak	4	9	10	8	8	13	6	9
End of epidemic week no.	15	19	17	17	19	19	18	17
Duration in weeks of epidemic	20	21	21	19	21	22	25	22
Duration in weeks of epidemic with at least 40% positive	11	8	12	b	10	11	9	13
Maximum weekly percent positive	51%	54%	57%	39%	55%	53%	53%	55%

a No two successive weeks of at least 10 specimens and a percent positive of at least 10%.

b No weeks with at least 10 specimens and a percent positive of at least 40%.

**Figure 4:**
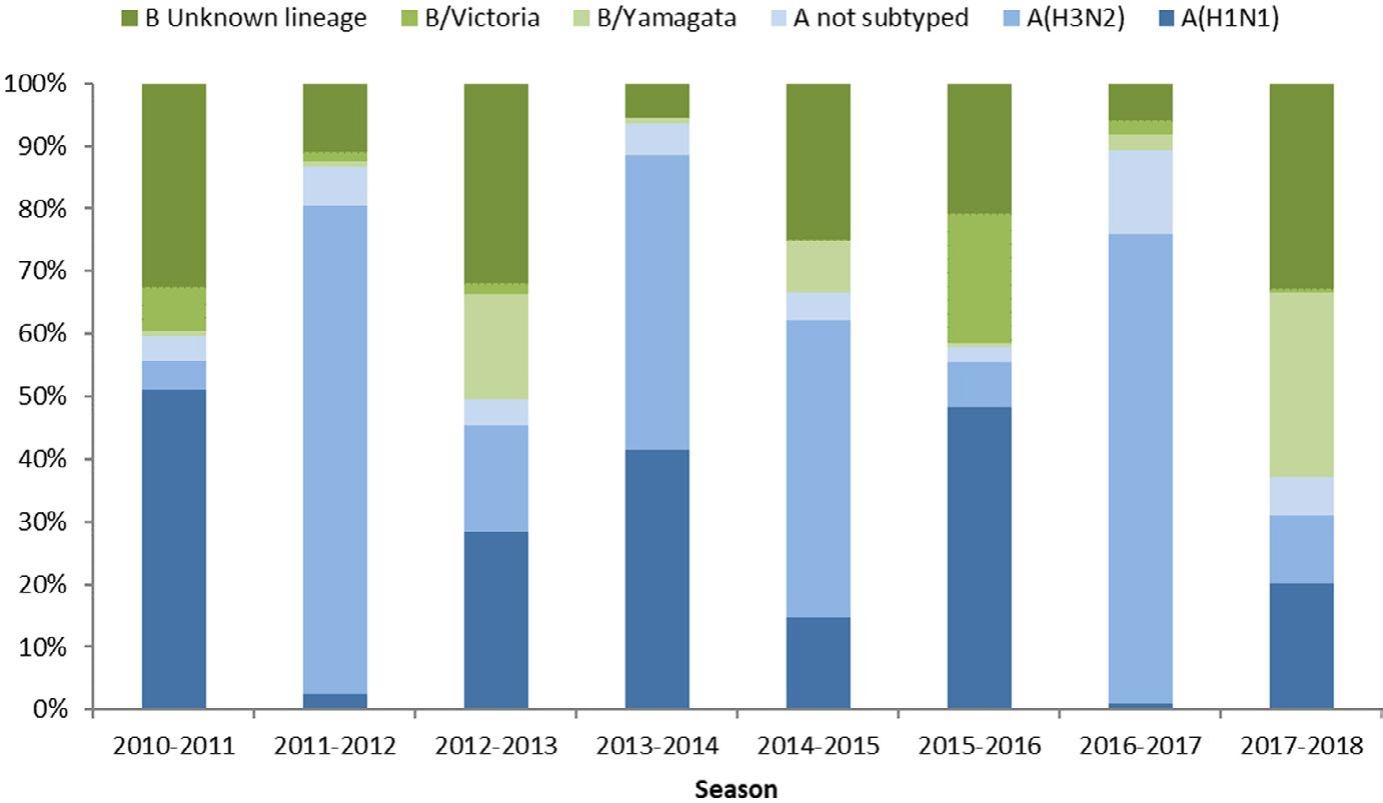
Distribution of influenza virus type, influenza A subtype and influenza B lineage from sentinel outpatient surveillance in the WHO European Region by season, 2010‐2018

The proportion of influenza A vs B viruses by season ranged from 94% A and 6% B in 2013‐2014 to 37% A and 63% B in 2017‐2018 (Table 1, Figure 4), while the proportion of subtyped A viruses that were A(H1N1)pdm09 vs A(H3N2) ranged from 92% A(H1N1)pdm09 and 8% A(H3N2) in 2010‐11 to 1% A(H1N1)pdm09 and 99% A(H3N2) in 2016‐17, and the proportion of B viruses assigned to a lineage that were from B/Yamagata vs B/Victoria ranged from 97% B/Yamagata and 3% B/Victoria in 2014‐2015 and 2017‐2018 to 4% B/Yamagata and 96% B/Victoria in 2015‐2016.

The dominant circulating influenza viruses in the Region were type A viruses in five seasons (2010‐2011, 2011‐2012, 2013‐2014, 2014‐2015, and 2016‐2017) and type B viruses in one season (2017‐2018), while both virus types were codominant in two seasons (2012‐2013 and 2015‐2016). Excluding the 2013‐2014 codominant A(H1N1)pdm09/(H3N2) season, A(H1N1)pdm09 and A(H3N2) virus subtypes alternated by year. The dominant influenza B virus lineage was B/Victoria in three seasons (2010‐2011, 2011‐2012, and 2015‐2016), B/Yamagata in four seasons (2012‐2013 to 2014‐2015, and 2017‐2018) and both were codominant in one season (2016‐2017).

For the first six seasons, at least 92% of influenza A viruses were subtyped; in 2016‐2017 and 2017‐2018, this decreased to no more than 85% (*P* < .001, chi‐squared test for Trend; Table 1). When proportion of A viruses not subtyped were calculated across seasons with dominant A virus subtype (ie, excluding 2013‐2014 data), there was a small but significant difference in percentage of A viruses not subtyped between A(H1N1)pdm09 and A(H3N2) dominant years; 9% (3437 of 39 874) in A(H1N1)pdm09 dominant years vs. 12% (3816 of 36 673) in A(H3N2) dominant years (*P* < .001, chi‐squared test; Table 1). The proportion of B viruses that were assigned a lineage increased over the study period from 19% in 2010‐2011 to 48% in 2017‐2018 (*P* < .001, chi‐squared test for Trend; Table 1).

The median start week of the epidemic using pooled European data was week 50 (range, week 46 [2016‐2017] to 51 [2011‐2012, 2013‐2014, 2014‐2015, and 2015‐2016]) (Table 2). The median peak of these epidemics was week 7 and the range was week 52 (2010‐2011 and 2016‐2017) to week 11 (2015‐2016); the median time from start to peak was 8.5 weeks and ranged from four (2010‐2011) to 13 weeks (2015‐2016). There was no significant correlation between the start and peak weeks distribution of virus types (*r* = .163; *P* = .700 and *r* = .096; *P* = .822, respectively) or influenza A subtypes (*r* = .039; *P* = .926 and *r* = .100; .814, respectively). The median end week of these epidemics was week 17.5, and the range was week 15 (2010‐2011) to week 19 (2014‐2015 and 2015‐2016); the median duration of the epidemic was 21 weeks and ranged from 19 (2013‐2014) to 25 weeks (2016‐2017). The median duration of at least 40% positive was 11 weeks, and the range was eight (2011‐2012) to 13 weeks (2017‐2018). There was no evidence of a linear change in week or duration for these temporal indicators over time. In the seven seasons when influenza B was detected in at least 10% of specimens for two successive weeks, B activity followed A activity temporally all years except in 2017‐2018 (simultaneous peak in week 50) and 2012‐2013 (B peak in week 49 and A peak in week 50).

Of 54 reporting entities, 37 (69%) had eight valid seasons based on at least 50 specimens and 20 weeks of reported data and 44 (81%) had five (Table S1). Among the 44, the median number of tested specimens, detections, and percent positive ranged from 70 (Latvia) to 5,241 (Spain), 34 (Latvia) to 2,686 (Spain), and 5% (Azerbaijan) to 58% (Austria), respectively. In any one season, there was variation across reporting entities in the distribution of virus type and influenza A subtype (Table 3); these distributions most closely aligned with the corresponding regional distributions when there was clear dominance of an influenza virus type or influenza A virus subtype. Of the six reporting entities that represented 48% of detections, only in Spain was an alternating influenza A subtype dominance pattern concurrent with that of the Region observed; the other 5 reporting entities each had three seasons with discordant seasonal dominant subtypes. In addition, there was variation in the distribution of these virological characteristics at the reporting entity level between seasons (Table S1).

**Table 3:** Distribution of reporting entity percentage of detections from sentinel outpatient surveillance that were type A viruses and percentage of subtyped A viruses that were A(H1N1)pdm09 in the WHO European Region by season, 2010‐2018

	No. of reporting entities (%)							
	2010‐2011	2011‐2012	2012‐2013	2013‐2014	2014‐2015	2015‐2016	2016‐2017	2017‐ 2018
Total no. of reporting entities	43 (100%)	42 (100%)	45 (100%)	43 (100%)	43 (100%)	42 (100%)	45 (100%)	47 (100%)
% of detections that were type A viruses (vs type B)								
0%‐19% type A	1 (2%)	(0%)	1 (2%)	(0%)	1 (2%)	(0%)	(0%)	10 (21%)
20%‐39% type A	4 (10%)	(0%)	11 (24%)	(0%)	6 (14%)	6 (14%)	(0%)	21 (45%)
40%‐59% type A	17 (41%)	3 (8%)	16 (36%)	2 (5%)	6 (14%)	14 (33%)	5 (11%)	14 (30%)
60%‐79% type A	13 (32%)	9 (23%)	11 (24%)	1 (2%)	20 (47%)	9 (21%)	8 (18%)	2 (4%)
80%‐100% type A	6 (15%)	28 (70%)	6 (13%)	38 (93%)	10 (23%)	13 (31%)	32 (71%)	0 (0%)
<10 total detections	2	2	0	2	0	0	0	0
% of subtyped A viruses that were A(H1N1)pdm09 (vs. type A(H3N2))								
0%‐19% A(H1N1)pdm09	(0%)	35 (92%)	5 (12%)	6 (15%)	25 (60%)	1 (3%)	42 (98%)	4 (10%)
20%‐39% A(H1N1)pdm09	1 (3%)	2 (5%)	5 (12%)	10 (25%)	7 (17%)	1 (3%)	1 (2%)	6 (14%)
40%‐59% A(H1N1)pdm09	1 (3%)	1 (3%)	9 (21%)	12 (30%)	5 (12%)	(0%)	(0%)	9 (21%)
60%‐79% A(H1N1)pdm09	3 (8%)	(0%)	12 (29%)	7 (18%)	3 (7%)	3 (8%)	(0%)	3 (7%)
80%‐100% A(H1N1)pdm09	32 (86%)	(0%)	11 (26%)	5 (13%)	2 (5%)	35 (88%)	(0%)	20 (48%)
<10 subtyped A viruses	6	4	3	3	1	2	2	5

**Figure 5:**
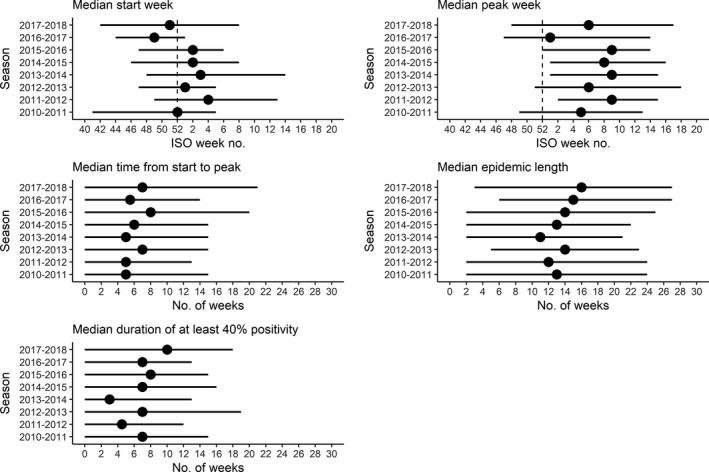
Median and range of start weeks, peak weeks and durations for reporting entity‐level influenza epidemics in the WHO European Region by season, 2010 to 2018. Based on data from reporting entity sentinel surveillance outpatient clinics. The dots represent the median value for a season and the ends of the lines indicate the minimum and maximum values. The division between calendar years is indicated by the dashed vertical line, where appropriate. ISO week 53 in 2015 is not represented in the median start and peak plots but is included in measures of duration

Across the Region, there was variation among reporting entities in the timing of epidemics within the same season (Figure 5). The seasonal reporting entities' median timings differed from regional timings, although similar broad patterns between the two across seasons were observed (Table 2 and Figure 5). There was also variation in the timing of reporting entity‐level epidemics between seasons (Table S1); a linear trend in timing of the start of the season (where a reporting entity had at least five valid seasons of data) was observed only in Portugal where there was a decrease (ie, earlier season start) over the study period (*P* = .008, *r *= −.847; linear regression). There was a weak positive association between longitude (in a west to east direction) and reporting entity‐level start of the epidemic in the 2010‐2011 season (*r* = .365; *P* = .019) and a moderate positive association in the 2012‐2013 (*r* = .484; *P* = .001), 2014‐2015 (*r* = .423; *P* = .006), and 2017‐2018 (*r* = .566; *P* < .001) season (Table 4). There was no evidence of an association between latitude and reporting entity‐level start of the epidemic for any season. Removing data from the Russian Federation did not alter the qualitative findings (Table S2).

**Table 4:** Associations between longitude and latitude of reporting entities in the WHO European Region and start of reporting entity epidemics by season, 2010‐2018

Direction	2010‐2011		2011‐2012		2012‐2013		2013‐2014		2014‐2015		2015‐2016		2016‐2017		2017‐ 2018	
	*P*	*r*	*P*	*r*	*P*	*r*	*P*	*r*	*P*	*r*	*P*	*r*	*P*	*r*	*P*	*r*
West to East	.019	.365	.403	.144	.001	.484	.303	.172	.006	.423	.358	.145	.553	−.094	<.001	.566
North to South	.880	−.024	.093	.284	.212	−.199	.202	.212	.564	−.093	.901	‐.020	.151	.226	.999	.000

Abbreviations: *P*, *P*‐value derived through linear regression; *r*, correlation coefficient.

When we restricted regional analysis to aggregated data up to the start of the epidemic, we found that the proportion of type A influenza viruses was greater compared to that using complete season data in 2014‐2015 and 2015‐2016 and less in 2010‐2011 and 2013‐2014 (*P* < .001, chi‐squared test) (Table S3). However, this affected the corresponding dominant virus type only in 2010‐11 (A dominant type with complete season sentinel outpatient clinic data versus A‐B codominant when considering start of the epidemic data only) and 2015‐2016 (A‐B codominant vs A). Among subtyped A viruses, the proportion of A(H3N2) was greater using data up to the start of the epidemics in the 2010‐2011, 2012‐2013 to 2015‐2016, and 2017‐18 seasons (*P* < .001, chi‐squared test), although this affected the corresponding dominant A subtype only in 2012‐2013 (A(H1N1) pdm09 vs A(H3N2)), 2013‐2014 (A(H1N1) pdm09‐A(H3N2) codominant vs A(H3N2)), and 2017‐2018 (A(H1N1)pdm09 vs A(H1N1)pdm09‐A(H3N2) codominant). Among B viruses ascribed to a lineage, the proportion of B/Victoria viruses was greater at the start of the epidemic in 2012‐2013 and the proportion of B/Yamagata viruses was greater in 2015‐2016 (*P* < .05, Fisher's exact test), although these differences did not affect the ultimate dominant B lineage determination. Virus data at the start of the epidemics were available between 24 (2012‐2013) and 34 (2015‐2016) reporting entities (predominantly in the West of the Region and Turkey and Israel). Total detections across the Region up to the start of the epidemic by season ranged from 276 (2016‐2017) to 529 (2014‐2015).

## 4 DISCUSSION

Over eight influenza seasons following the 2009 pandemic, we characterized influenza virus detections from sentinel surveillance in outpatients and found that annual epidemics of influenza in the European Region exhibited some patterns. For the first seven seasons, influenza A viruses were dominant or codominant, and excluding the 2013‐2014 codominant season, A(H1N1)pdm09 and A(H3N2) virus dominance alternated each year, a pattern that was absent in similar regional data prior to the pandemic.^11^ There was no evidence that the distribution of virus type or A virus subtypes correlated with epidemic start or peak timing indicators. In 2017‐2018, the pattern was disrupted when influenza B viruses dominated and the Yamagata lineage was more frequently detected than influenza A subtypes.

The alternating A virus subtype pattern observed in the European Region was not consistently observed at the individual reporting entity‐level (only Spain among the six reporting entities that provided half of all detections), nor was it observed elsewhere in the northern hemisphere (Canada or the USA). In three seasons of dominant A(H1N1)pdm09 viruses in the European Region (2010‐2011, 2012‐2013, and 2017‐2018), A(H3N2) viruses were dominant in both Canada^12^, ^13^, ^14^ and the USA^15^, ^16^, ^17^ and in 2013‐2014 during which the A virus subtypes were codominant, A(H1N1)pdm09 viruses were dominant in Canada^18^ and the USA.^19^ The dominant virus distributions reported from the Region matched that of contemporaneous influenza epidemics in Canada and/or the USA during four seasons (2011‐2012,^14^, ^20^ 2014‐2015,^21^, ^22^ 2015‐2016,^23^, ^24^ and 2016‐2017^25^, ^26^) for A virus subtype.

Except for the 2012‐2013 and 2017‐2018 epidemics in which at least 50% of the viruses were influenza B, within a season increases in influenza A virus activity preceded increases in influenza B virus activity. We found no linear relationship in any temporal characteristics of these epidemics or number of specimens or detections. For most seasons, the maximum weekly percent positive in specimens from outpatients was approximately 55% and most seasons had approximately 10 weeks of substantially increased (≥40% positivity) virus activity (the 2013‐2014 season was a notable exception with no weeks of ≥40% positivity).

We found evidence of a west‐to‐east spread across the Region for half of the seasons under consideration and this pattern has been observed previously when using pooled sentinel and non‐sentinel (specimens taken for diagnostic purposes and derived from various sources including outpatient sites that are not part of sentinel networks, hospitals, outbreak investigations, long‐term care homes, and closed facilities) data.^11^ North‐to‐south spread across the Region has also been identified previously but this was not apparent in our analysis.

While we found that the proportional distribution of viruses using cumulative data up to the start of the epidemic was often different than analysis at the end of the season (though perhaps this is not surprising as late season increases of B are often observed), virus dominance using cumulative data up to the start of the epidemic was most often concurrent with dominant viruses for cumulative data to the end of the season. Based on a subset of reporting entities, this early information is useful to inform risk assessments which in turn can prepare others.^27^ These findings may be helpful for early season forecasting efforts.

Seasons with a dominant influenza B virus were rare (2017‐2018 only) and this is consistent with global findings.^28^ Nevertheless, influenza B virus circulates every year and often in significant numbers. In a study of hospitalized adults in the United States, influenza B caused similar severity to influenza A,^29^ and it has been shown to cause fatal illness in persons of all ages.^30^, ^31^ Primarily trivalent influenza vaccines are used in Europe^32^; however, given that both B lineages might circulate every year, recently reporting entities have begun to examine the cost‐effectiveness of using quadrivalent vaccine.^33^ This became a particularly relevant discussion after the 2017‐18 season, when the B component of the vaccine was a different lineage than the dominant circulating one. These surveillance data also highlight the need to train and communicate the need for more B lineage testing which is still relatively infrequent.

We observed wide variation in virus proportions between reporting entities and this might be due to a variety of factors such as population susceptibility based on prior vaccinations and exposure to prior circulating viruses as well as contact patterns within and between reporting entities. Notably, only Portugal experienced progressively earlier starts to their epidemics over the study period; there were no apparent systematic changes to surveillance practices which might account for this observation (personal communication). There was no linear relationship to duration of the regional epidemics over the study period, which is in contrast to findings using pooled sentinel and non‐sentinel data over 20 years.^34^

These data have several limitations, most notably that there is great variation between reporting entities in the number of detections and percent positive by reporting entity within a season, likely reflecting differing approaches and capacity of reporting entity sentinel surveillance systems (including population coverage, sampling approach, and case definition) or different epidemiological situations in the reporting entities (virus distributions and timing of reporting entity‐level epidemics varied within a given season). It is possible that the regional data are biased by those countries submitting the majority of detection data and approaches to weighting reporting entity data for future similar analysis could be considered.

We have analyzed reporting entity defined sentinel surveillance data but it is possible that for some reporting entities diagnostic virological data from non‐sentinel sources (with no systematic sampling scheme) have been erroneously included or sentinel surveillance systems had limited or no systematic sampling. In addition, in some sentinel systems, there may be non‐systematic sampling and we are unable to stratify the virological data from these systems by presentation with ILI or ARI. Other approaches to define the epidemic period (eg, moving epidemic method^35^ based on syndromic or combined syndromic and virological data) are often used by reporting entities and this may result in different reporting entity published estimates than those presented here. Subtype, lineage, and dominant type determination only used those tested in the denominator, and it is possible that the distribution of untested is different. During the 2009 influenza pandemic, some laboratories introduced a PCR assay to detect A(H1N1)pdm09 virus and specimens testing negative but identified as influenza A were reported as A unsubtyped. Although the correct testing algorithm includes subtyping A viruses, some laboratories, mainly clinical, ceased subtyping for A(H3N2). This is likely to have resulted in an increased proportion of non‐subtyped A viruses particularly in A(H3N2) dominant seasons, which might bias the results and highlights the need to understand reporting entity‐level practices. Finally, we were not able to describe age‐specific trends as the data were not available by age group.

Our findings highlight the substantial diversity between seasonal influenza epidemics in terms of virus distribution and level of activity, underscoring the challenges to accurately predicting the impact of a forthcoming influenza epidemic based on retrospective data in a large region.^36^ Nevertheless, it is important to understand the nature of influenza epidemics at this level to help healthcare professionals at European and reporting entity‐level target prevention and control strategies and ensure capacity to respond. We show that early data during a season are likely to portend virus dominance, which can be used to target risk communication and public health action. Furthermore, monitoring circulating influenza viruses is critical for tracking changes and assessing their match with vaccine strains, and for adapting vaccination and antiviral treatment strategies.

## DECLARATIONS

The authors alone are responsible for the views expressed in this publication and they do not necessarily represent the views, decisions, or policies of the institutions with which they are affiliated. Maps and terminology used in this publication do not imply any opinions on the part of ECDC and WHO or its partners about the legal status of the countries and territories shown or about their borders.

## APPENDIX 1

### Members of the WHO European Region and European Influenza Surveillance network

Albania: Artan Simaku and Iris Hasibra (Hatibi); Armenia: Liana Torosyan and Shushan Sargsyan; Austria: Monika Redlberger‐Fritz and Judith H. Aberle; Azerbaijan: Oleg Salimov and Nazakat Abdullayeva; Belarus: Veronika Shimanovich and Natalia Gribkova; Belgium: Nathalie Bossuyt and Isabelle Thomas; Bulgaria: Anna Kurchatova and Neli Korsun; Croatia: Goranka Petrović and Vladimir Draženović; Cyprus: Maria Koliou and Despo Pieridou; Czech Republic: Martina Havlickova and Jan Kyncl; Denmark: Tyra Grove Krause and Ramona Trebbien; Estonia: Olga Sadikova and Natalja Kuznetsova; France: Vincent Enouf and Sibylle Bernard‐Stoecklin; Finland: Niina Ikonen and Outi Lyytikäinen; Georgia: Ann Machablishvili and Khatuna Zakhashvili; Germany: Silke Buda and Ralf Dürrwald; Greece: Ourania Kalkouni and Georgia Gioula; Hungary: Zsuzsanna Molnár and Mónika Rózsa; Ireland: Linda Dunford and Joan O Donnell; Iceland: Gudrun Sigmundsdottir and Gudrun Erna Baldvinsdottir; Israel: Zalman Kaufman and Michal Mandelboim; Italy: Caterina Rizzo and Maria Rita Castrucci; Kazakhstan: Sultanova Meirim and Altynay Sagimbay; Kyrgyzstan: Otorbaeva Dinagul and Bodoshev Azat; Latvia: Raina Nikiforova and Natalija Zamjatina; Lithuania: Asta Skrickienė and Algirdas Griškevičius; Luxembourg: Guillaume Fournier and Joel Mossong; Malta: Jackie M. Melillo and Graziella Zahra; Montenegro: Božidarka Rakočević and Zoran Vratnica; Netherlands: Gé Donker and Adam Meijer; North Macedonia: Golubinka Bosevska and Vladimir Mikikj; Norway: Trine Hessevik Paulsen and Olav Hunges; Poland: Lidia B. Brydak and Katarzyna Luniewska; Portugal: Raquel Guiomar and Ana Paula Rodrigues; Republic of Moldova: Stefan Gheorghita and Constantin Spinu; Romania: Rodica Popescu and Alina Ivanciuc; Russian Federation: Anna A. Sominina and Elena Burtseva; Serbia (excluding Kosovo^*^): Dragana Dimitrijevic and Svetlana Filipovic‐Vignjevic; Kosovo^*^: Arijana Kalaveshi; Slovenia: Maja Sočan and Katarina Prosenc; Slovakia: Ivan Bakoss and Edita Staroňová; Spain: Amparo Larrauri and Francisco Pozo, on behalf of the Spanish Influenza Surveillance System; Sweden: AnnaSara Carnahan and Mia Brytting; Switzerland: Damir Perisa and Ana Rita Gonçalves; Tajikistan: Tamanno Safarova and Niginamo Zakirova; Turkey: Emine Avci and Ayse Basak Altas; Ukraine: Oksana Artemchuk and Iryna Demchishyna; United Kingdom by reporting entity: England: Richard Pebody and Joanna Ellis: Northern Ireland: Conall McCaughey and Mark O'Doherty: Scotland: Jim Mcmenamin and Arlene Reynolds: Wales: Simon Cottrell and Catherine Moore; Uzbekistan: Sultana Djemileva and Ravshan Rakhimov; WHO Collaborating Centre London: John McCauley and Rod Daniels.
