# Supplementary material for: Alternating patterns of seasonal influenza activity in the WHO European Region following the 2009 pandemic, 2010‐2018
Source: Influenza Other Respir Viruses. 2020 Jan 16;14(2):150–61. doi: 10.1111/irv.12703 (PMC7040975; doi:10.1111/irv.12703)
Supplement: Supplementary file 1 [file IRV-14-150-s001.docx]

**Supplementary Table 1.** Summary statistics of virological distributions and timing characteristics of reporting-entity level influenza epidemics using sentinel outpatient surveillance data, 2010/11 to 2017/18 seasons^a^

| **Reporting-entity** | **Longitude**^8^ | **Latitude**^8^ | **No. of valid seasons ^b^** | **Median (range) no. of specimens** | **Median (range) no. of detections** | **Median (range) % positive** | **Median (range) % of detections that were type A viruses** | **Median (range) % of subtyped A viruses that were A(H1N1)^c^** | **Median (range) start week of epidemic** | **Median (range) peak week of epidemic** | **Median (range) epidemic length** |
| --- | --- | --- | --- | --- | --- | --- | --- | --- | --- | --- | --- |
| Albania | 41.0 | 20.0 | 8 | 659 (271-1046) | 150 (68-338) | 29 (18-37) | 75 (22-100) | 62 (0-100) | 2 (48-5) | 7 (49-12) | 10 (8-16) |
| Armenia | 40.0 | 45.0 | 6 | 875 (52-1549) | 161 (9-627) | 22 (13-58) | 41 (22-100) | 100 (1-100) | 51 (46-7) | 6 (47-17) | 19 (12-27) |
| Austria | 47.2 | 13.2 | 8 | 555 (335-843) | 325 (124-513) | 58 (37-61) | 78 (32-99) | 58 (0-98) | 2 (50-4) | 6 (51-14) | 12 (11-16) |
| Azerbaijan | 40.3 | 47.3 | 8 | 524 (68-1168) | 43 (0-97) | 5 (0-25) | 29 (3-100) | 0 (0-0) | 2 (50-6) | 3 (51-14) | 10 (9-10) |
| Belarus | 53.0 | 28.0 | 8 | 1375 (957-2242) | 117 (29-322) | 9 (3-14) | 72 (50-95) | 47 (0-100) | 4 (50-14) | 9 (4-15) | 14 (3-18) |
| Belgium | 50.5 | 4.0 | 8 | 823 (581-1405) | 414 (247-817) | 53 (34-61) | 67 (34-100) | 59 (0-100) | 49 (46-2) | 6 (51-12) | 17 (13-21) |
| Bosnia and Herzegovina | 44.0 | 18.0 | 0 | N/A | N/A | N/A | N/A | N/A | N/A | N/A | N/A |
| Bulgaria | 43.0 | 25.0 | 8 | 250 (195-421) | 76 (30-111) | 29 (15-41) | 80 (9-100) | 77 (0-92) | 3 (49-5) | 7 (1-11) | 6 (4-11) |
| Croatia | 45.1 | 15.3 | 3 | 3749 (1195-5854) | 1794 (0-2747) | 47 (0-48) | 66 (40-91) | 29 (11-47) | 48 (48-48) | 52 (48-4) | 22 (21-22) |
| Cyprus | 35.0 | 33.0 | 0 | N/A | N/A | N/A | N/A | N/A | N/A | N/A | N/A |
| Czech Republic | 49.5 | 15.3 | 8 | 457 (298-630) | 198 (19-249) | 38 (6-49) | 70 (30-84) | 38 (1-98) | 50 (44-14) | 8 (4-15) | 18 (2-25) |
| Denmark | 56.0 | 10.0 | 8 | 295 (116-471) | 139 (20-214) | 43 (17-55) | 58 (17-99) | 47 (1-100) | 2 (49-6) | 6 (3-12) | 9 (8-16) |
| Estonia | 59.0 | 26.0 | 8 | 512 (348-897) | 189 (125-265) | 35 (26-43) | 84 (54-100) | 36 (0-100) | 3 (49-6) | 7 (52-11) | 13 (10-18) |
| Finland | 64.0 | 26.0 | 8 | 416 (237-1127) | 133 (62-446) | 32 (14-53) | 81 (40-94) | 39 (0-100) | 2 (46-4) | 6 (51-9) | 13 (7-20) |
| France | 46.0 | 2.0 | 8 | 3924 (2643-5451) | 1640 (1315-2640) | 48 (34-58) | 67 (29-100) | 54 (1-96) | 50 (42-2) | 8 (51-11) | 20 (17-27) |
| Georgia | 42.0 | 43.3 | 8 | 413 (199-1029) | 106 (17-334) | 26 (9-37) | 57 (0-96) | 50 (0-100) | 3 (46-6) | 7 (50-18) | 14 (10-18) |
| Germany | 51.0 | 9.0 | 8 | 3800 (1692-5654) | 1395 (242-2242) | 36 (11-50) | 71 (32-94) | 42 (1-99) | 51 (49-5) | 8 (4-12) | 17 (15-23) |
| Greece | 39.0 | 22.0 | 6 | 291 (209-582) | 144 (79-353) | 49 (32-71) | 71 (15-99) | 51 (0-99) | 3 (2-5) | 5 (4-8) | 11 (8-14) |
| Hungary | 47.0 | 20.0 | 8 | 940 (663-1948) | 333 (102-472) | 33 (14-46) | 70 (15-95) | 60 (0-99) | 3 (51-6) | 12 (51-14) | 13 (11-15) |
| Iceland | 65.0 | -18.0 | 0 | N/A | N/A | N/A | N/A | N/A | N/A | N/A | N/A |
| Ireland | 53.0 | -8.0 | 8 | 918 (509-1585) | 464 (186-874) | 48 (37-55) | 64 (29-98) | 29 (0-98) | 48 (43-1) | 7 (52-14) | 19 (16-24) |
| Israel | 31.3 | 34.5 | 8 | 1529 (1075-1916) | 514 (300-864) | 35 (24-45) | 65 (39-98) | 32 (0-98) | 52 (41-3) | 5 (1-9) | 15 (13-23) |
| Italy | 42.5 | 12.5 | 8 | 1867 (990-2783) | 864 (297-1492) | 48 (30-61) | 64 (30-96) | 39 (0-95) | 50 (46-1) | 6 (2-12) | 17 (14-20) |
| Kazakhstan | 48.0 | 68.0 | 8 | 712 (338-1051) | 161 (50-260) | 22 (11-30) | 64 (52-96) | 35 (0-77) | 2 (46-4) | 6 (1-9) | 12 (6-23) |
| Kyrgyzstan | 41.0 | 75.0 | 8 | 351 (65-982) | 66 (0-400) | 20 (0-41) | 47 (12-100) | 21 (0-100) | 51 (45-4) | 5 (48-10) | 10 (5-16) |
| Latvia | 57.0 | 25.0 | 7 | 70 (62-105) | 34 (24-51) | 50 (23-63) | 73 (33-100) | 0 (0-0) | 4 (3-11) | 4 (4-11) | 3 (2-5) |
| Lithuania | 56.0 | 24.0 | 8 | 513 (104-1005) | 327 (30-607) | 54 (29-72) | 79 (15-100) | 35 (2-100) | 2 (49-7) | 5 (3-15) | 13 (9-18) |
| Luxembourg | 49.5 | 6.1 | 8 | 615 (308-1123) | 286 (83-722) | 46 (27-64) | 62 (22-99) | 56 (0-100) | 1 (49-4) | 6 (52-13) | 14 (9-16) |
| Malta | 35.5 | 14.4 | 1 | 58 (58-58) | 45 (45-45) | 78 (78-78) | 82 (82-82) | 0 (0-0) | 5 (5-5) | 5 (5-5) | 5 (5-5) |
| Montenegro | 42.3 | 19.2 | 0 | N/A | N/A | N/A | N/A | N/A | N/A | N/A | N/A |
| Netherlands | 52.3 | 5.5 | 8 | 624 (179-785) | 270 (16-451) | 43 (9-57) | 67 (26-96) | 51 (1-98) | 49 (48-8) | 7 (3-9) | 17 (9-20) |
| North Macedonia | 41.5 | 22.0 | 2 | 204 (67-341) | 88 (18-158) | 37 (27-46) | 64 (28-100) | 50 (0-100) | 4 (4-4) | 6 (6-6) | 8 (8-8) |
| Norway | 62.0 | 10.0 | 8 | 321 (249-418) | 147 (71-194) | 44 (29-55) | 76 (28-99) | 42 (0-94) | 51 (46-3) | 4 (50-8) | 11 (7-15) |
| Poland | 52.0 | 20.0 | 8 | 1265 (459-1627) | 442 (26-704) | 34 (6-45) | 66 (28-99) | 85 (0-100) | 2 (50-11) | 10 (2-16) | 13 (4-16) |
| Portugal | 39.3 | -8.0 | 8 | 396 (136-973) | 173 (49-473) | 43 (32-56) | 74 (33-100) | 62 (0-100) | 52 (44-6) | 4 (49-11) | 12 (5-18) |
| Republic of Moldova | 47.0 | 29.0 | 8 | 495 (187-781) | 113 (45-394) | 24 (18-55) | 67 (13-100) | 78 (0-94) | 4 (48-10) | 9 (51-15) | 12 (7-25) |
| Romania | 46.0 | 25.0 | 8 | 443 (253-893) | 148 (80-252) | 33 (25-56) | 78 (25-100) | 57 (0-97) | 2 (52-3) | 8 (1-13) | 12 (9-15) |
| Russian Federation | 60.0 | 100.0 | 8 | 1670 (1443-2012) | 248 (114-485) | 14 (8-24) | 75 (63-95) | 40 (0-86) | 3 (49-8) | 9 (1-10) | 15 (12-20) |
| Serbia | 44.0 | 21.0 | 8 | 326 (107-631) | 170 (49-308) | 52 (43-72) | 88 (22-100) | 54 (0-100) | 4 (49-11) | 8 (50-13) | 11 (2-17) |
| Slovakia | 48.4 | 19.3 | 8 | 264 (120-390) | 98 (26-243) | 42 (11-62) | 78 (14-100) | 52 (0-100) | 5 (51-13) | 10 (3-13) | 8 (2-10) |
| Slovenia | 46.1 | 14.5 | 8 | 561 (414-796) | 273 (198-383) | 47 (45-56) | 61 (46-100) | 45 (0-99) | 52 (50-2) | 8 (5-12) | 15 (10-18) |
| Spain | 40.0 | -4.0 | 8 | 5241 (4478-6034) | 2686 (2136-3507) | 51 (45-58) | 65 (24-99) | 50 (0-98) | 49 (44-51) | 5 (52-12) | 22 (19-26) |
| Sweden | 62.0 | 15.0 | 8 | 1196 (815-1677) | 337 (190-495) | 26 (18-35) | 67 (23-98) | 40 (1-97) | 51 (48-2) | 9 (4-15) | 17 (14-19) |
| Switzerland | 47.0 | 8.0 | 8 | 972 (580-1304) | 476 (194-748) | 49 (33-57) | 63 (30-98) | 52 (2-97) | 50 (48-2) | 7 (52-13) | 19 (13-20) |
| Tajikistan | 39.0 | 71.0 | 2 | 168 (141-194) | 59 (56-62) | 36 (32-40) | 53 (15-91) | 68 (68-68) | 5 (2-8) | 11 (9-12) | 7 (3-11) |
| Turkey | 39.0 | 35.0 | 8 | 3175 (2060-4958) | 735 (414-1503) | 22 (14-37) | 69 (45-98) | 60 (0-93) | 50 (48-5) | 2 (1-11) | 18 (12-24) |
| Turkmenistan | 40.0 | 60.0 | 0 | N/A | N/A | N/A | N/A | N/A | N/A | N/A | N/A |
| Ukraine | 49.0 | 32.0 | 5 | 516 (384-2599) | 66 (38-506) | 17 (9-19) | 53 (22-100) | 25 (0-100) | 4 (48-9) | 10 (49-11) | 12 (3-16) |
| United Kingdom^d^ | - | - | - | - | - | - | - | - | - | - | - |
| England | 54.0 | -2.0 | 7 | 2300 (1662-4581) | 625 (244-1674) | 23 (14-37) | 71 (33-100) | 34 (0-99) | 49 (47-4) | 8 (52-11) | 17 (13-22) |
| Northern Ireland | 54.0 | -2.0 | 8 | 209 (138-412) | 78 (15-228) | 37 (11-55) | 71 (44-100) | 12 (0-100) | 1 (50-3) | 7 (50-14) | 11 (10-17) |
| Scotland | 54.0 | -2.0 | 8 | 1531 (525-2383) | 361 (101-738) | 22 (7-54) | 55 (29-91) | 14 (0-98) | 51 (45-8) | 10 (2-13) | 20 (8-23) |
| Wales | 54.0 | -2.0 | 7 | 105 (61-232) | 59 (7-91) | 34 (9-56) | 77 (27-100) | 14 (0-98) | 51 (51-4) | 6 (5-9) | 10 (10-11) |
| Uzbekistan | 41.0 | 64.0 | 6 | 218 (134-302) | 60 (16-118) | 28 (11-44) | 57 (36-100) | 8 (0-97) | 3 (2-8) | 7 (2-12) | 9 (6-14) |

^a^Based on virological data from sentinel ILI or ARI outpatient surveillance sources. Data for Kosovo^[[1]](#footnote-2)^* are not presented

^b^At least 50 specimens from ILI/ARI cases and 20 weeks of data. Some of these seasons did not reach criteria for start season and so fewer included in the range and median presented for the timing vars.

^c^ Season percentages of A(H1N1) were not calculated where less than 10 detections in total. As such, the number of values in the median and range may be less than the number of valid seasons for a given reporting-entity. ^d^UK longitude and latitude coordinates used for England, Northern Ireland, Scotland and Wales

**Supplementary Table 2.** Associations between longitude and latitude of reporting-entities in the WHO European Region (excluding the Russian Federation) and start of reporting-entity epidemics by season, 2010-2011 to 2017-2018

| **Direction** | **2010-2011** | | **2011-2012** | | **2012-2013** | | **2013-2014** | | **2014-2015** | | **2015-2016** | | **2016-2017** | | **2017- 2018** | |
| --- | --- | --- | --- | --- | --- | --- | --- | --- | --- | --- | --- | --- | --- | --- | --- | --- |
|  | **p** | **r** | **p** | **r** | **p** | **r** | **p** | **r** | **p** | **r** | **p** | **r** | **p** | **r** | **p** | **r** |
| West to East | 0.022 | 0.360 | 0.713 | 0.064 | 0.003 | 0.461 | 0.302 | 0.174 | <0.001 | 0.573 | 0.428 | 0.127 | 0.382 | -0.140 | <0.001 | 0.584 |
| North to South | 0.734 | -0.055 | 0.139 | 0.2552 | 0.104 | -0.261 | 0.225 | 0.204 | 0.661 | -0.072 | 0.813 | -0.038 | 0.160 | 0.223 | 0.814 | -0.036 |

r: correlation coefficient

p: p-value derived through linear regression

**Supplementary Table 3.** Summary of regional aggregated virological data from sentinel ILI or ARI outpatient sentinel surveillance sources at the start of the epidemic^a^ by season, 2010-2011 to 2017- 2018

|  | **2010-2011** | **2011-2012** | **2012-2013** | **2013-2014** | **2014-2015** | **2015-2016** | **2016-2017** | **2017- 2018** |
| --- | --- | --- | --- | --- | --- | --- | --- | --- |
| Specimens tested | 6,761 | 9,724 | 7,241 | 9,659 | 9,131 | 9,289 | 5,876 | 7,869 |
| Influenza detections | 333**^b^** | 308**^c^** | 293**^d^** | 440**^e^** | 529**^f^** | 374**^g^** | 276**^h^** | 427**^i^** |
| **Virus distribution^j^** | | | | | | | | |
| **Influenza A** | **168 (50%)** | **276 (90%)** | **138 (47%)** | **392 (89%)** | **404 (76%)** | **264 (71%)** | **242 (88%)** | **159 (37%)** |
| A(H1N1)pdm09 | 124 (78%) | 7 (3%) | 43 (35%) | 132 (34%) | 70 (19%) | 201 (80%) | 3 (1%) | 65 (47%) |
| A(H3N2) | 36 (23%) | 245 (97%) | 80 (65%) | 252 (66%) | 303 (81%) | 51 (20%) | 231 (99%) | 74 (53%) |
| Subtype distribution vs. complete season data (p value) | p<0.001 | p=0.778 | p<0.001 | p<0.001 | p=0.014 | p<0.001 | p=0.935 | p<0.001 |
| A not subtyped | 8 | 24 | 15 | 8 | 31 | 12 | 8 | 20 |
| **Influenza B** | **165 (50%)** | **32 (10%)** | **155 (53%)** | **48 (11%)** | **125 (24%)** | **110 (29%)** | **34 (12%)** | **268 (63%)** |
| B/Yamagata | 1 (8%) | 5 (-^k^) | 41 (80%) | 8 (-^k^) | 44 (92%) | 5 (14%) | 2 (20%) | 113 (97%) |
| B/Victoria | 11 (92%) | 1 (-^k^) | 10 (20%) | 1 (-^k^) | 4 (8%) | 31 (86%) | 8 (80%) | 4 (3%) |
| Lineage distribution vs. complete season data (p value) | p>0.999**^l^** | p=0.052**^l^** | p=0.033**^l^** | p>0.999**^l^** | p=0.133**^l^** | p=0.019**^l^** | p=0.052**^l^** | p=0.824**^l^** |
| Unknown lineage | 153 | 26 | 104 | 39 | 77 | 74 | 24 | 151 |
| Type distribution vs. complete season data (p value) | p<0.001 | p=0.125 | p=0.404 | p<0.001 | p<0.001 | p<0.001 | p=0.424 | p=0.935 |
| **Dominant virus type** | A/B | A | A/B | A | A | A | A | B |
| **Dominant A virus subtype** | A(H1N1)pdm09 | A(H3N2) | A(H3N2) | A(H3N2) | A(H3N2) | A(H1N1)pdm09 | A(H3N2) | A(H1N1)pdm09/A(H3N2) |
| **Dominant B virus lineage** | B/Victoria | **-** | B/Yamagata | **-** | B/Yamagata | B/Victoria | B/Victoria | B/Yamagata |

^a^As defined by the first of two successive weeks of at least 10 specimens and a percent positive of 10%

^b^% of detections by reporting-entity (n=30): United Kingdom (England) (22.2%), France (16.5%), Israel (13.8%), Spain (12.9%), Belgium (4.5%), Georgia (4.5%), Ukraine (2.7%), United Kingdom (Scotland) (2.7%), Czech Republic (2.1%), Finland (2.1%), and less than 2% from each of 20 reporting-entities.

^c^% of detections by reporting-entity (n=25): Spain (27.9%), Turkey (15.9%), France (10.1%), Italy (9.1%), Israel (6.5%), Ireland (4.5%), Sweden (4.2%), Czech Republic (3.6%), Belgium (2.6%), Serbia (2.6%), United Kingdom (England) (2.3%) and less than 2% from each of 14 reporting-entities.

^d^% of detections by reporting-entity (n=24): France (27.6%), United Kingdom (England) (16%), Germany (8.2%), Spain (8.2%), United Kingdom (Scotland) (7.8%), Ireland (5.1%), Turkey (4.4%), Belgium (3.8%), Czech Republic (2%), Israel (2%), Italy (2%), Netherlands (2%) and less than 2% from each of 12 reporting-entities.

^e^% of detections by reporting-entity (n=28): Turkey (28.4%), Spain (22.3%), France (13.6%), Sweden (6.8%), Israel (5.2%), United Kingdom (England) (3.6%), Ireland (2.5%), United Kingdom (Scotland) (2.3%), Belgium (2%), Italy (2%) and less than 2% from each of 18 reporting-entities.

^f^% of detections by reporting-entity (n=32): Spain (24%), Italy (9.1%), France (8.3%), Belgium (6.2%), United Kingdom (Scotland) (6%), Germany (5.7%), United Kingdom (England) (5.3%), Azerbaijan (4.5%), Netherlands (4.2%), Portugal (3.6%), Uzbekistan (2.6%), Israel (2.1%), Sweden (2.1%), Switzerland (2.1%) and less than 2% from each of 18 reporting-entities.

^g^% of detections by reporting-entity (n=34): Turkey (9.9%), Israel (9.4%), Germany (7%), Spain (7%), United Kingdom (England) (7%), Ireland (6.7%), France (5.3%), United Kingdom (Scotland) (5.3%), Belgium (5.1%), Kazakhstan (4.8%), Sweden (4.5%), Norway (4%), Czech Republic (3.5%), Netherlands (3.5%), Portugal (2.9%), Finland (2.4%), Kyrgyzstan (2.1%) and less than 2% from each of 17 reporting-entities.

^h^% of detections by reporting-entity (n=26): Spain (20.7%), Kyrgyzstan (13%), Portugal (12.7%), France (11.6%), Armenia (7.6%), Kazakhstan (4%), Norway (4%), Germany (3.6%), United Kingdom (Scotland) (3.3%), Israel (2.9%), Ireland (2.2%), United Kingdom (England) (2.2%) and less than 2% from each of 14 reporting-entities.

^i^% of detections by reporting-entity (n=30): Spain (18.7%), Croatia (9.8%), Turkey (9.6%), France (9.1%), United Kingdom (Scotland) (6.8%), Germany (5.9%), Israel (5.4%), Italy (5.2%), United Kingdom (England) (4.9%), Ireland (3.7%), Tajikistan (3%), Portugal (2.8%), Armenia (2.1%), United Kingdom (Northern Ireland) (2.1%) and less than 2% from each of 16 reporting-entities.

^j^ For influenza virus type percentage calculations, the denominator was total detections; for A virus subtype and B virus lineage, it was total influenza A viruses subtyped and total influenza B viruses with lineage determined, respectively.

^k^Percentages were not calculated where less than 10 detections

^l^Fisher’s Exact Test. All other p-values were derived using the Chi-squared Test.

1. * In accordance with Security Council resolution 1244 (1999). [↑](#footnote-ref-2)
